# Respiratory Syncytial Virus Exacerbates OVA-mediated asthma in mice through C5a-C5aR regulating CD4^+^T cells Immune Responses

**DOI:** 10.1038/s41598-017-15471-w

**Published:** 2017-11-09

**Authors:** Xinyue Hu, Xiaozhao Li, Chengping Hu, Ling Qin, Ruoxi He, Lisha Luo, Wei Tang, Juntao Feng

**Affiliations:** 1 Department of Respiratory Medicine (Department of Respiratory and Critical Care Medicine), Key Cite of National Clinical Research Center for Respiratory Disease, Xiangya Hospital, Central South University, Changsha, Hunan 410008 China; 2 Department of Nephrology, Xiangya Hospital, Central South University, Changsha, Hunan 410008 China

## Abstract

Asthma exacerbation could be induced by respiratory syncytial virus (RSV), and the underlying pathogenic mechanism is related to complement activation. Although complement might regulate CD4^+^T cells immune responses in asthma model, this regulation existed in RSV-induced asthma model remains incompletely characterrized. In this study, we assessed the contribution of C5a-C5aR to CD4^+^T cell immune responses in RSV-infected asthma mice. Female BALB/C mice were sensitized and challenged with ovalbumin (OVA) while treated with RSV infection and C5a receptor antagonist (C5aRA) during challenge period. RSV enhanced lung damage, airway hyperresponsiveness, and C5aR expressions in asthma mice, while C5aRA alleviated these pathologic changes. The percentages of Th1, Th2 and Th17 cells were increased, while the percentage of Treg cells was decreased in RSV-infected asthma mice compared with asthma mice. IFN-γ, IL-4, IL-10 and IL-17A levels have similar trend with Th1, Th2, Th17 and Treg cells. Notably, above changes of CD4^+^T cells and their related cytokines were reversed by C5aRA. Together, the data indicates that RSV infection could apparently increase C5a and C5aR expression in the pathogenesis of RSV-infected asthma mice, meanwhile C5aRA prevents some of the CD4^+^T cells immune changes that are induced by RSV.

## Introduction

Asthma exacerbation is a major reason for hospitalization, meanwhile asthma patients with frequent exacerbations have a significantly greater long-term decline in pulmonary function^1,2^. RSV, influenza and parainfluenza viruses are important triggers of acute asthma exacerbations^3^. The pathology of RSV-induced asthma exacerbation model involves increased inflammation and cytokine levels, increased airway hyperresponsiveness (AHR) and a cellular immune response comprising lymphocytes, eosinophils and neutrophils^4^.

As a critical component of lymphocytes, CD4^+^T cells refer to various asthma immunological processes^5^. Traditionally, asthma has been viewed as a Th2 disease with increased IgE levels and eosinophilc inflammation contributing to AHR^6^, while evidence from an asthma mouse model suggests Th1 cells also modulate the disease^7^. Lately, Th17 cells were found to contribute to airway inflammation and AHR^8^.

Recently, C3a and C5a is viewed as proinflammatory mediators via binding to C3aR and C5aR separately that recruits inflammatory cells and participates in T cells regulation^9,10^. Supporting above notion that complement plays a vital role in T cells regulation, inhibition of C5aR1 signaling in human CD4^+^T cells, absent C3aR and C5aR siganling in mouse CD4^+^T cells, mediates generation of Foxp3^+^T regulatory cells and suspends effctor fuctions^11–13^. Moreover, C3a and C5a is identified as novel potential therapeutic target for asthma. Studies show that C3a and C5a regulates allergic sensitization and affects dendritic cell-T cell interctions^14–17^. In addition, C5a receptor antagonist (C5aRA) reduces airway inflammatory cells and cytokine responses in an asthma mouse model^18^. C3a receptor (C3aR) deficiency mice infected with RSV do not develop AHR and accelerate viral clearance, meanwhile reduce mucus, IL-17A, tachykinin production and lung inflammation compared with wild-type^19^. C3a-C3aR signaling in the context of acute RSV infection results in activation of the IL-17A pathway^20^. C3aR and C5aR protein expression is increased in fatal asthma compared to individuals who died from nonpulmonary causes and bronchial biopsy samples from individuals with mild intermittent asthma^21^. What is more, complement activation is involved in the pathogenic mechanisms of virus-related diseases, including RSV, H1N1, influenza virus and H7N9 virus, by increasing the production of pro-inflammatory mediators^19,22,23^. However, little is known about whether or not C5a-C5aR could be activated by RSV, and there is seldom report about the regulatory mechanisms between C5a-C5aR and CD4^+^T cells in RSV-induced asthma exacerbation.

Elucidation of the C5a-C5aR activated by RSV infection and the contribution of C5a-C5aR to CD4^+^T cell immunity is of significance in RSV-induced asthma exacerbation. The objective of this study is to clarify the relationship between C5a-C5aR and CD4^+^T cells in RSV-infected asthma mice.

## Materials and Methods

### Mice

Six-week-old female BALB/c mice (20 ± 2 g) were purchased from the Experimental Animal Center of Central South University (Changsha, Hunan, China). All animals were maintained in specific pathogen-free conditions at an appropriate temperature and humidity. All animal experiments were managed according to the guideline of the Experimental Animal Center of Central South University. All experimental protocols were approved by the Ethics Committee in Xiangya Hospital of Central South University, including any relevant details.

### Animal models

Forty-eight mice were randomly divided into eight groups (n = 6 per group): control mice (Control, control mice for RSV group and asthma group respectively), mice infected with RSV for 7d (RSV 7d), C5aRA-treated RSV-infected mice (C5aRA-RSV); asthma mice; RSV-infected asthma mice (RSV-asthma), C5aRA-treated asthma mice (C5aRA-asthma), C5aRA-treated RSV-asthma mice (C5aRA-RSV-asthma).

For the RSV infection mouse model, mice were inoculated with ~10^6^ PFU of RSV (A2 strain) in 50 μl endotoxin-free PBS by intranasal instillation and intraperitoneal (i.p). injection under isofluorane anesthesia on days 0 to 2. For C5aRA treatment of the RSV-infection mice, C5aRA (0.3 ug/mouse, W54011, Abcam) was pretreated by caudal vein injection on day-1 before RSV infection described above. The control group received an equal amount of PBS. Mice were sacrificed on day 7.

The asthma mouse model was developed as previously^24^. Briefly, mice were sensitized by i.p. injection of 20 μg OVA (Grade V, Sigma) and 2 mg of aluminum hydroxide (Pierce, Rockford, IL) in 200 μl phosphate buffered saline (PBS) (pH 7.4) on days 0, 7 and 14. Mice were challenged by repeated exposure to OVA (5%) from a PARIBOYN (085G1205) nebulizer (PARI GmbH, Starnberg, Germany) driven by compressed air at 20 L/min for 30 min from day 21 to 27. For RSV-infected asthma mice, asthma mice were inoculated with RSV before OVA challenge on days 21, 22 and 23. For C5aRA treatment of the asthma and RSV-infected asthma mice, mice were treated with C5aRA (0.3 ug/mouse, W54011, Abcam) by caudal vein injection one hour before challenge from day 21 to 27. The control group received an equal amount of PBS. Mice were sacrificed on day 28. All mice were terminated after collection of samples for analyses.

### Airway resistance

Mice were anesthetized with pentobarbital (125 mg/kg i.p.) and intubated. Animals were ventilated with 100% oxygen at a rate of 150 breaths per min and a tidal volume of 0.25 ml at 3 cm H_2_O-positive end-expiratory pressure. After a steady baseline was established, animals were administered graded doses of methacholine (0.3125, 0.625, 1.56 and 3.125 mg/ml) by aerosol, and changes in airway resistance were recorded as cm H_2_O × seconds per milliliter^19^.

### Inflammatory cells in bronchoalveolar lavage fluid (BALF)

To assess accumulation of inflammatory cells in BALF, mice were sacrificed with pentobarbital after measuring airway resistance. In each animal, the trachea was cannulated, and the left bronchi were tied for histological analysis. Then, the right air lumen was washed three times with 0.8 ml of PBS. Recovered BALF was centrifuged at 1200 *rpm* for 5 min at 4 °C. The cell pellet was resuspended in cell preservation liquid. The leukocyte count in the cell pellet was determined from a smear using Wright’s stain. Under a microscope, a minimum of 300 cells were counted and classified as lymphocytes, neutrophils or eosinophils based on morphological criteria. Data was assessed by pathologists who are proficient in pathologic analysis.

### Histological analysis

Left lungs were fixed in 4% neutral-buffered formalin, dissected, embedded in paraffin, and sectioned to 3-μm. Sections were stained with hematoxylin-eosin (HE) and periodic acid-Schiff (PAS) and examined with light microscopy for evidence of lung damage and goblet cell metaplasia.

Immunohistochemistry (C5aR, CD3, CD4 and LY6G protein) and immunoflourescence (RSV fusion protein) were performed on paraffin-embedded sections. For both protocols, serial 3-μm thick sections of lung were mounted on Superfrost Plus slides, dewaxed, and rehydrated with an alcohol gradient and PBS. Antigen retrieval was performed with citrate (pH 6.0). Endogenous peroxidase was blocked with 3% H_2_O_2_ in water for 20 min, and nonspecific binding was blocked with diluted normal rabbit serum for 60 min. For immunohistochemistry, sections were incubated with C5aR primary antibody (ab117579, Abcam), CD3 primary antibody (32416, SAB), CD4 primary antibody (44038, SAB) and LY6G primary antibody (GB11229, Guge) respectively for 18 h at 4 °C. Labeling was identified using the SP goat IgG kit (PV-6001, ZSGB-Bio), according to the manufacturer’s instructions. The chromogenic reaction solution contained 3,3′-diaminobenzidine (DAB) (ZLI-9018, ZSGB-Bio), and counterstaining was performed with Mayer’s hematoxylin. For immunofluorescence, sections were incubated with RSV fusion protein primary antibody (SC-57998, Santa Cruz) for 16 h at 4 °C. Labeling was identified with goat anti mouse IgG-FITC secondary antibody (SC-2010, Santa Cruz). Normal rabbit and normal rat sera were used in control group for immunohistochemistry and immunofluorescence respectively.

### Isolation of lymphocytes from lung tissue

Lung tissues were excised completely and minced in serum-free RPMI 1640 medium under aseptic conditions and incubated with 0.4 mg/ml collagenase IV (LS004186, Worthington) and 1U/ml DNase I (Roche) for 3 h at 37 °C. Cell suspensions were filtered through a series of nylon meshes ranging from 70-mm to 40-mm and washed with PBS. Lymphocyte-enriched cell suspension was acquired by percoll density gradient (70% and 30%, GE Healthcare) centrifugation. Cells were stained for flow cytometric analyses.

### Flow cytometry

Isolated lymphocytes were divided equally between tubes, and nonspecific binding was blocked with normal mouse serum (Sigma). To differentiate T-cells, lymphocytes were surface-stained with anti-CD3 (APC, Cat.17-0032, eBioscience) and anti-CD4 (FITC, Cat.11-0042, eBioscience) for 30 minutes at 4 °C in the dark. Staining of intracellular cytokines was performed as previously described^25^. Briefly, lymphocytes were suspended in RPMI 1640 (Gibco) with 10% fetal bovine serum (FBS) and activated by incubation with phorbol 12-myristate 13-acetate (PMA, 50ng/ml, P8139, Sigma) and ionomycin (1 mg/ml, I3909, Sigma) at 37 °C and 5% CO_2_ for 5 hours. Brefeldin A (3 mg/ml, Cat.004506, eBioscience) was added after 30 minutes of incubation. Lymphocytes were washed and stained with cell surface markers and permeabilized with Cytofix/Cytoperm. Intracellular cytokines were identified using anti-mouse IFN-γ (PE, Cat.12-7311, eBioscience), anti-mouse IL-4 (PE, Cat.12-7041, eBioscience), and anti-mouse IL-17A (PE, Cat.17-7177, eBioscience); rat IgG1 K Isotype Control (PE, Cat.12-4301, eBioscience) was used to detect non-specific binding by antigen-specific antibody. For foxp3 staining, lymphocytes were surface-stained with anti-CD4 (FITC, Cat.11-0042, eBioscience) and anti-CD25 (APC, Cat.17-0251, eBioscience) for 30 minutes at 4 °C in the dark and permeabilized with Cytofix/Cytoperm (eBioscience). Lymphocytes were stained with anti-mouse foxp3 antibody (PE, Cat.12-4771, eBioscience) for 45 minutes; rat IgG2a K Isotype Control (PE, Cat.12-4321, eBioscience) was used to detect non-specific binding by antigen-specific antibody.

Lymphocytes were analyzed with a Becton Dickinson FACSCalibur™ platform using Cell Quest software. Percentages of CD3+CD4+T cells, including Th1 cells: CD3^+^CD4^+^IFN-γ^+^; Th2 cells: CD3^+^CD4^+^IL-4^+^; Th17 cells: CD3^+^CD4^+^IL-17A^+^; and Treg cells: CD4^+^CD25^+^Foxp3^+^ were calculated.

### Cytokines in BALF, serum, and lung tissue

Serum and BALF were diluted with PBS, and lung samples were prepared by homogenization in PBS-containing protease inhibitors (Complete, Roche Diagnostics). Commercially available ELISA kits were used to quantify IFN-γ (BMS606, eBioscience), IL-4 (BMS613, eBioscience), IL-10 (BMS614/2, eBioscience), IL-17A (BMS6001, eBioscience), and C5a (ELM-CCC5A-1, Ray biotech) in BALF, serum, and lung tissues, according to the manufacturers’ instructions.

### Statistical analysis

Data is expressed as mean ± SEM. Statistical analyses for multiple groups were performed with one-way analysis of variance (ANOVA), and between-group comparisons were evaluated with the least significant difference (LSD) t-test (Prism software; Graphpad). Significance was assumed at P < 0.05.

## Results

### RSV aggravates while C5aRA reduces lung damage, inflammatory cells infiltration in BALF and airway resistance in RSV-infected asthma mice

**Figure 1:**
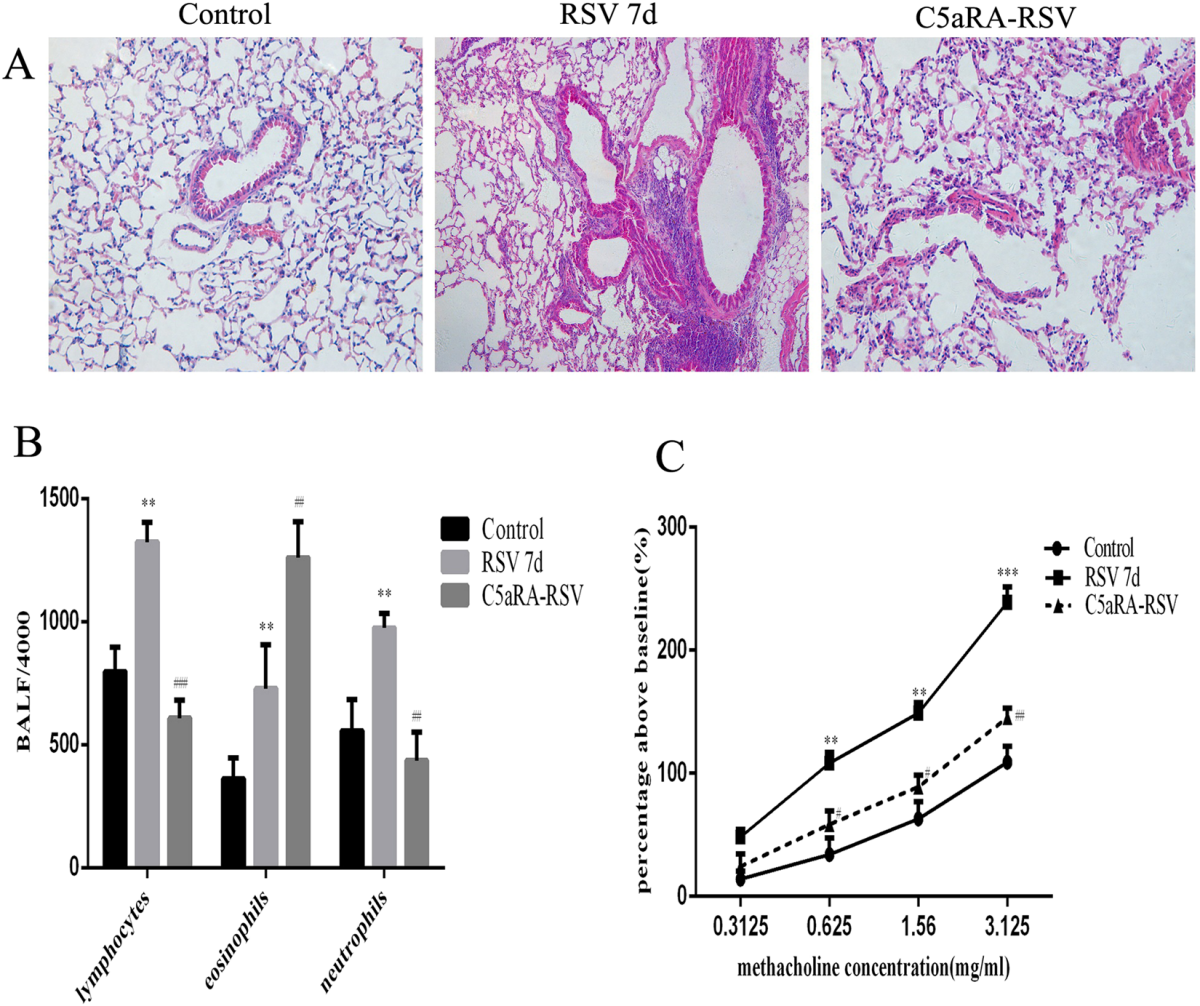
The impact of C5aRA in RSV-infected mice. (**A**) Representative images of H&E staining (100x). (**B**) Inflammatory cell infiltration in BALF; (**C**) AHR. ^*^P < 0.05, ^**^P < 0.01, ^***^P < 0.001 vs. control; ^#^P < 0.05, ^##^P < 0.01, ^###^P < 0.001 vs. RSV 7d.

The association of complement activation with RSV-induced AHR was investigated in a well-characterized mouse model of RSV infection. Compared with control mice, RSV-infected mice had interstitial edema, infiltration of inflammatory cells, and thickened alveolar walls; while these pathological changes were prevented by pretreatment with C5aRA (Fig. 1A).

**Figure 2:**
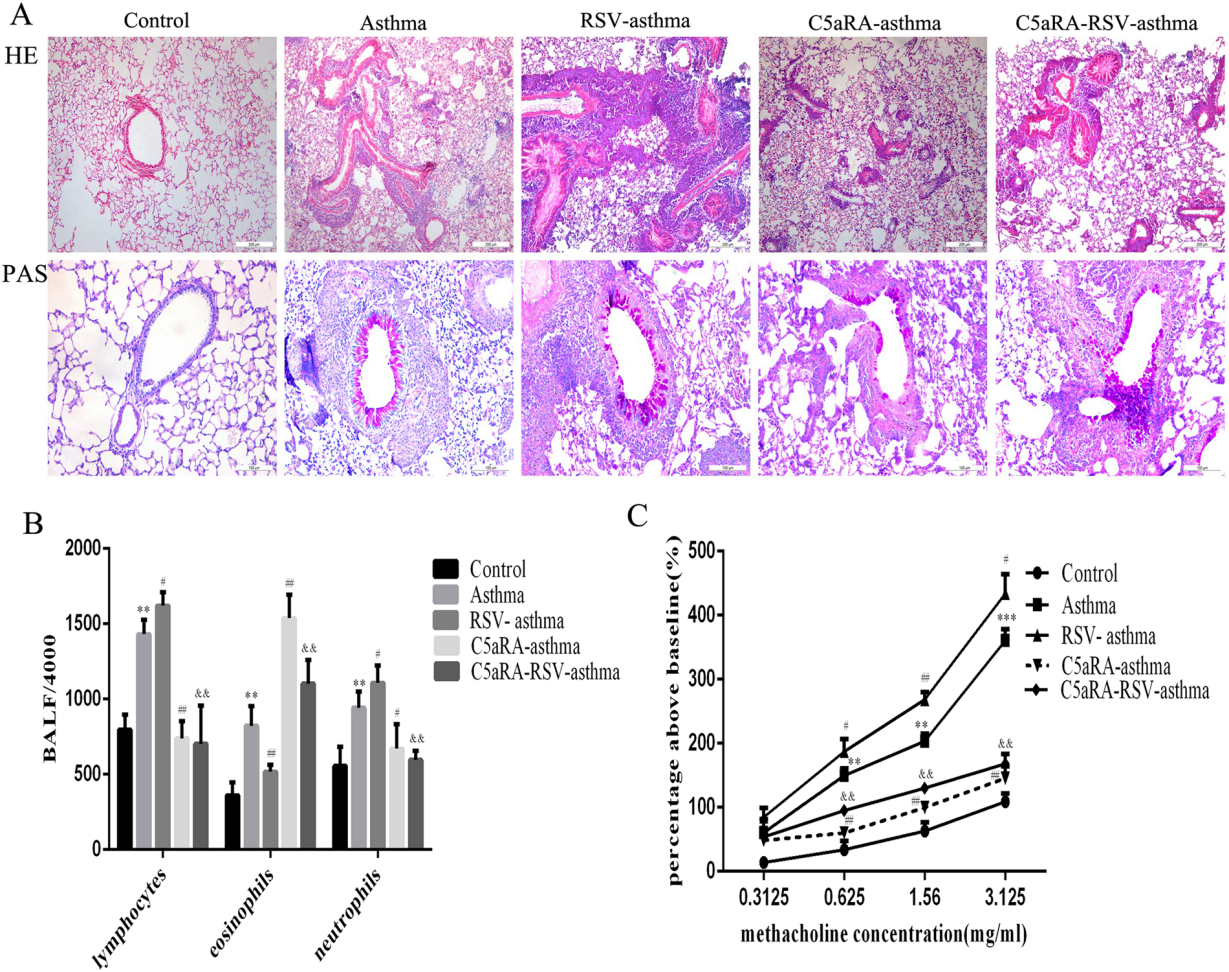
C5aRA reduces the impact of RSV infection on lung damage, inflammatory cell infiltration in BALF, and airway resistance in asthma mice. (**A**) Representative images of H&E staining (100x) and PAS staining (200x). (**B**) Inflammatory cell infiltration in BALF; (**C**) AHR. ^*^P < 0.05, ^**^P < 0.01, ^***^P < 0.001 vs. control; ^#^P < 0.05, ^##^P < 0.01, ^###^P < 0.001 vs. asthma; ^&^P < 0.05, ^&&^P < 0.01, ^&&&^P < 0.001 vs. RSV-asthma.

In addition, the role of C5aRA in RSV-infected asthma mice was explored. As Fig. 2A shown, histologic analyses of lung tissues indicated that airways were thickened, airway lumens were narrowed, and there were mucous cells and goblet cells metaplasia and more infiltration of inflammatory cells around blood vessels and bronchi in asthma mice compared with control mice. These damages described above were further exacerbated in RSV-infected asthma mice, but alleviated in C5aRA-treated asthma mice and C5aRA-treated RSV-infected asthma mice.

The number of lymphocytes, neutrophils and eosinophils in BALF was remarkably increased in RSV-infected mice and asthma mice compared with control mice and significantly increased in RSV-infected asthma mice compared with asthma mice. Interestingly, the number of lymphocytes and neutrophils in BALF was significantly decreased in C5aRA-treated asthma mice and C5aRA-treated RSV-infected asthma mice compared with asthma mice and RSV-infected asthma mice respectively, but number of eosinophils increased (Figs 1B and 2B).

Airway resistance was added with methacholine administration in a dose-dependent manner in all mice. There were evident increases in airway resistance in RSV-infected mice and asthma mice compared with control mice. Moreover, airway resistance was more severe in RSV-infected asthma mice compared with asthma mice. However, C5aRA could prevent some of the airway hyperresponsiveness in RSV-infected mice, asthma mice and RSV-infected asthma mice (Figs 1C and 2C).

To testify the infiltrating cells in different groups, CD3, CD4 and LY6G protein expressions were tested by immunohistochemistry. As the results shown, the expressions of CD3 and CD4 protein were much more obvious than LY6G protein in all groups. The expressions of CD3 and CD4 protein were increased in RSV-infected mice and asthma mice compared with control mice. Furthermore, RSV infection could augment CD3 and CD4 expressions in RSV-infected asthma mice. However, changes described above in RSV-infected mice, asthma mice and RSV-infected mice were reversed by C5aRA to some extent. LY6G expressions were no significant differences among the different grous (Supplementary Figure 1).

### C5aRA reduces RSV burden in the lung tissues of RSV-related mice

**Figure 3:**
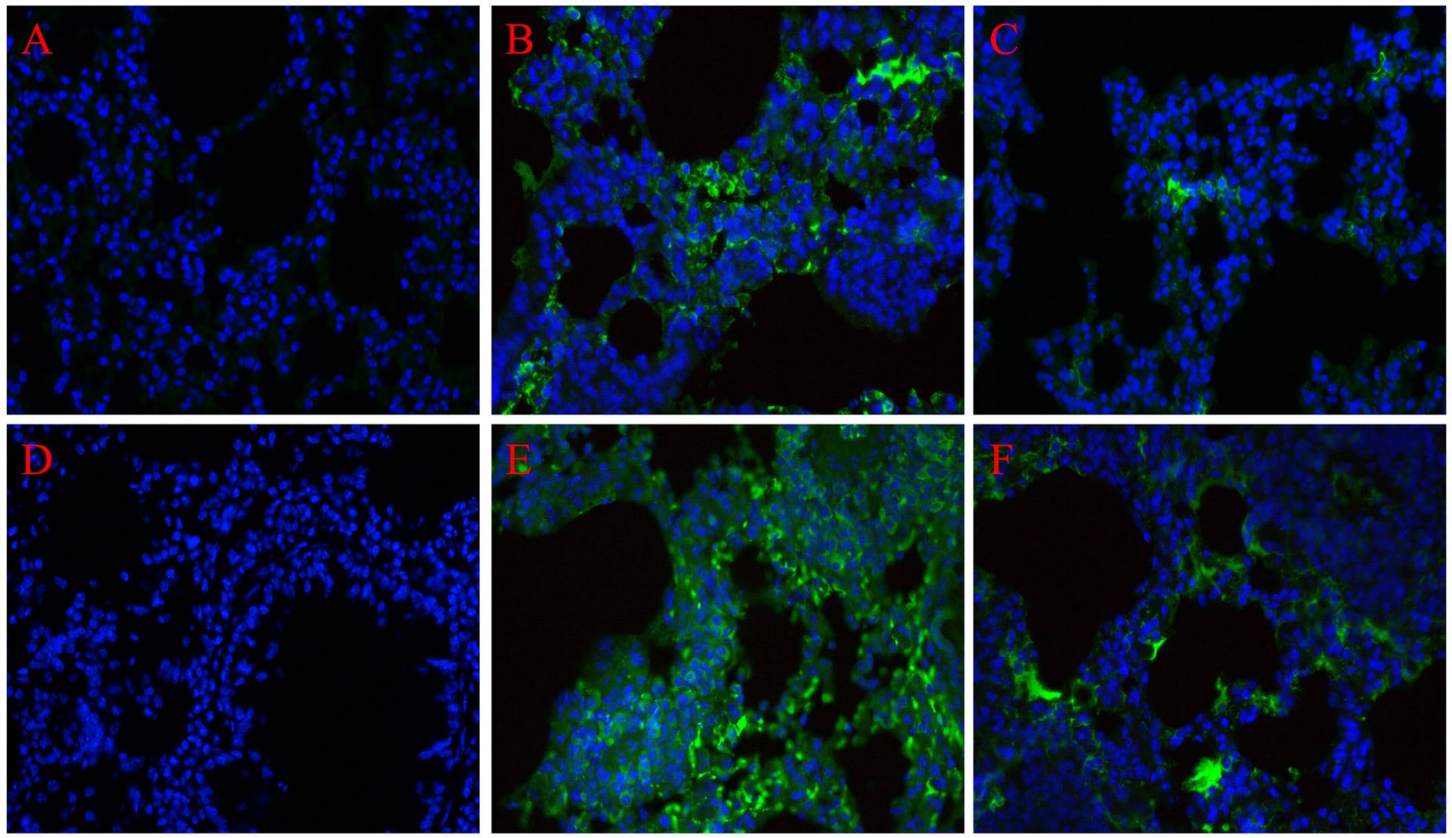
C5aRA reduces RSV burden in the lung tissues of RSV-infected mice. Representative images of immunofluorescence staining for RSV fusion protein in lung tissues (400×). (**A**) Control; (**B**) RSV 7d; (**C**) C5aRA-RSV; (**D**) asthma; (**E**) RSV-asthma; (**F**) C5aRA-RSV-asthma. Blue, cell nucleus; light green, RSV fusion protein.

To further explore the impact of C5aRA in RSV burden change in the lung tissues of RSV-infected mice and RSV-infected asthma mice, immunofluorescence staining for RSV fusion protein was used. As Fig. 3 shown, RSV fusion protein mainly deposited in interstitial infiltration area in RSV-infected mice and RSV-infected asthma mice while RSV fusion protein levels were further increased in RSV-infected asthma mice compared with RSV-infected mice. Nevertheless, C5aRA treatment both alleviated RSV deposition in RSV-infected mice and RSV-infected asthma mice.

### RSV raises C5a and C5aR expression in RSV-infected asthma mice

**Figure 4:**
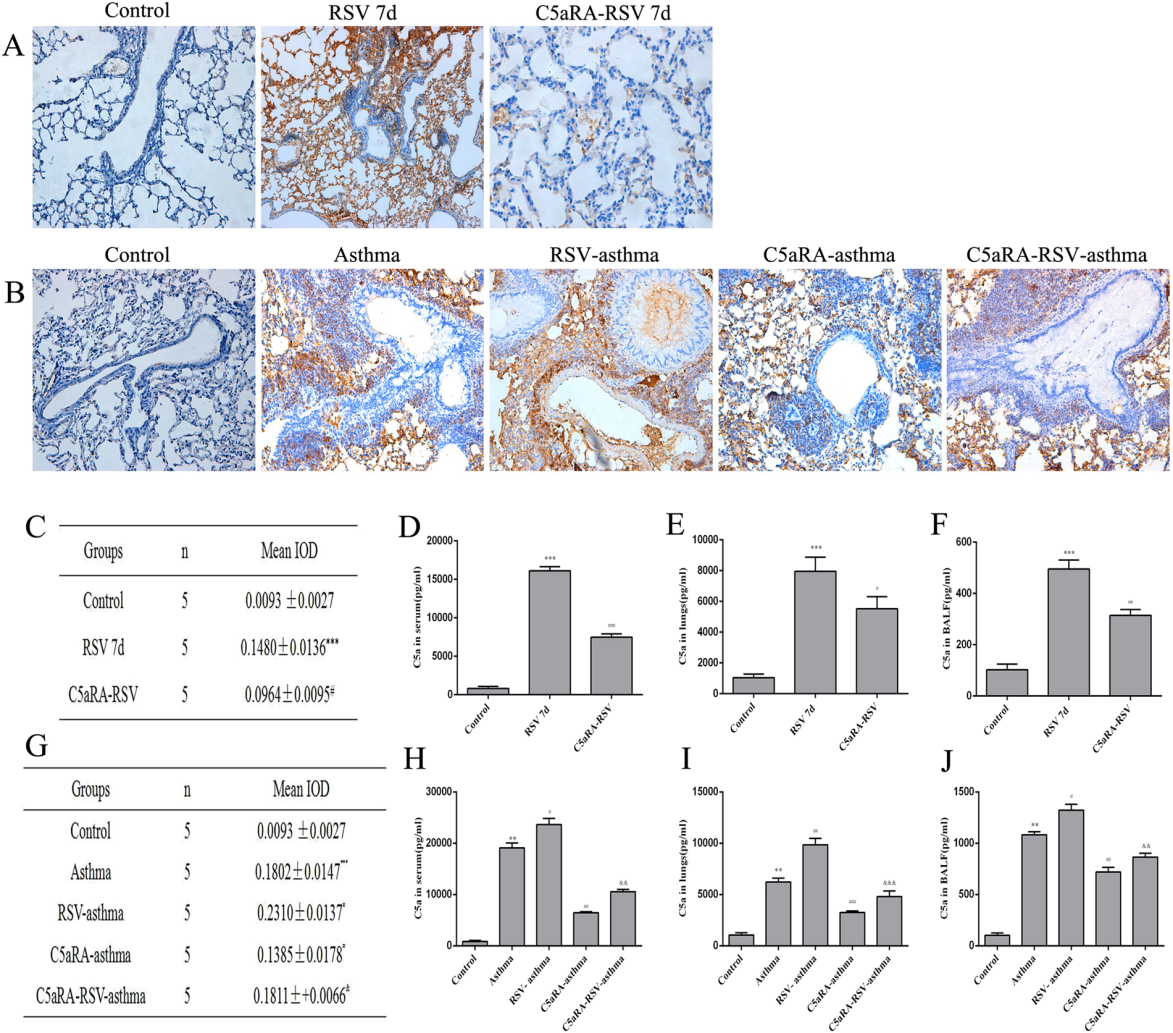
The expressions of C5aR protein and levels of C5a in different mice. (**A**) Representative images of immunohistochemistry staining for C5aR protein in lung tissues of control, RSV and C5aRA-RSV mice (200x). (**B**) Representative images of immunohistochemistry staining for C5aR protein in lung tissues of control, asthma, RSV-asthma, C5aRA-asthma and C5aRA-RSV-asthma mice (200x). (**C**) The semiquantitative of C5aR protein expressions in lung tissues of control, RSV and C5aRA-RSV mice. (**D**–**F**) Serum C5a levels (**D**), Lung C5a levels (**E**) and BALF C5a levels (**F**) in control, RSV and C5aRA-RSV mice. (**C**–**F**) ^*^P < 0.05, ^**^P < 0.01, ^***^P < 0.001 vs. control; ^#^P < 0.05, ^##^P < 0.01, ^###^P < 0.001 vs. RSV 7d. (**G**) The semiquantitative of C5aR protein expressions in lung tissues of control, asthma, RSV-asthma, C5aRA-asthma and C5aRA-RSV-asthma mice. (**H**–**J**) Serum C5a levels (**H**), Lung C5a levels (**I**) and BALF C5a levels (**J**) in control, asthma, RSV-asthma, C5aRA-asthma and C5aRA-RSV-asthma mice. (**G**–**J**) ^*^P < 0.05, ^**^P < 0.01, ^***^P < 0.001 vs. control; ^#^P < 0.05, ^##^P < 0.01, ^###^P < 0.001 vs. asthma; ^&^P < 0.05, ^&&^P < 0.01, ^&&&^P < 0.001 vs. RSV-asthma.

C5aR expressions were located in the submucosa, airway epithelium and inflammatory cells in RSV-infected mice and asthma mice but with no expression in control mice. C5aR levels appeared higher in the lung tissues of RSV-infected asthma mice compared with asthma mice. However, C5aR expressions in lung tissues were significantly lower in RSV-infected mice, asthma mice and RSV-infected asthma mice treated with C5aRA. Furthermore, to describe C5a level changes in different groups, ELISA was used to detect C5a level in BALF, serum and lung tissues. As the results shown, the tendency of C5a levels were in accordance with C5aR protein expressions (Fig. 4).

### RSV disturbs while C5aRA overcomes the balance of CD4^+^T cells proportions and relevant cytokines

**Figure 5:**
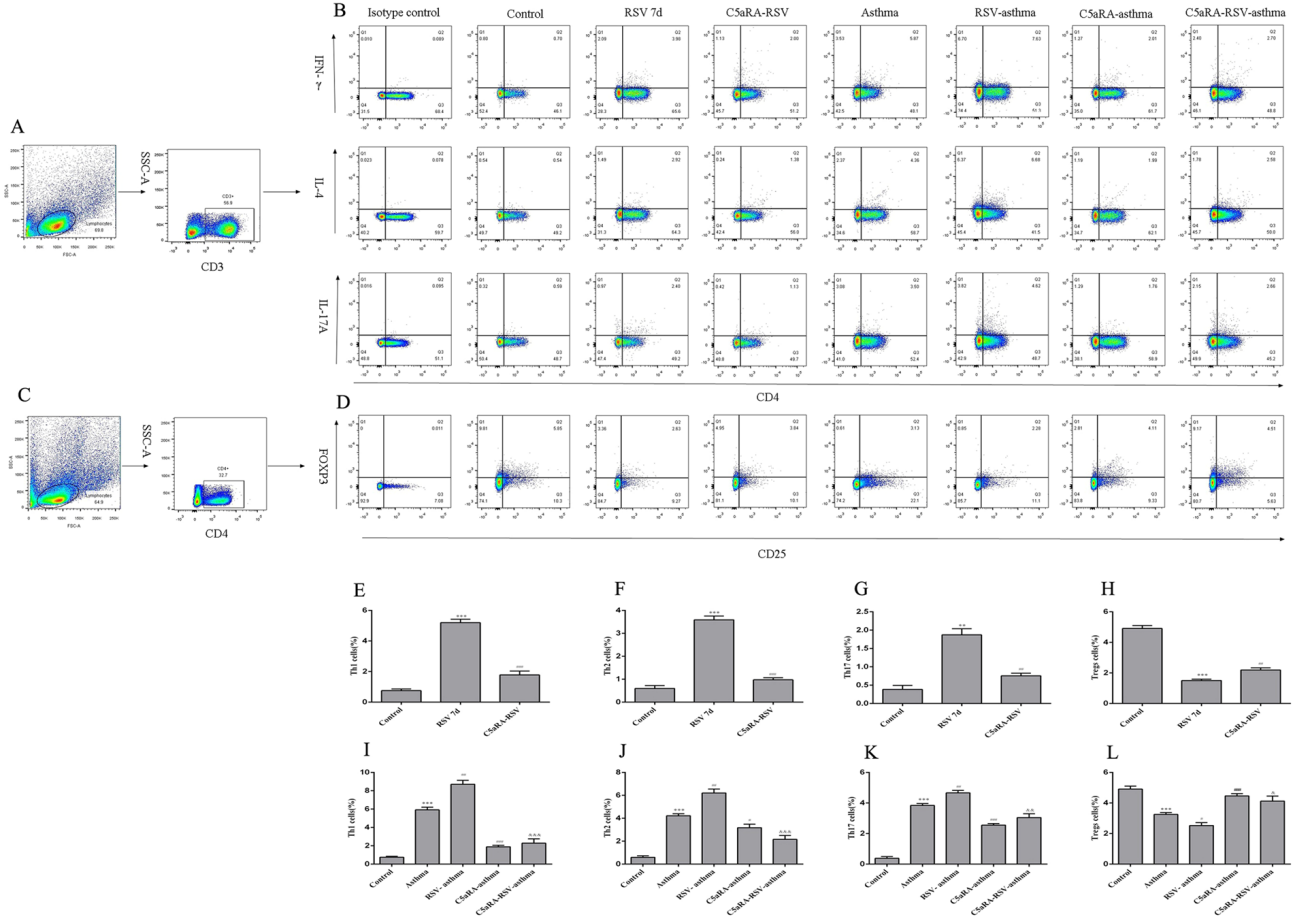
Percentages of Th1, Th2, Th17 and Treg cells in lung tissues. (**A**) Th1, Th2, Th17 cells within CD4^+^T cells were identified based on their expression of CD3^+^. (**B**) Representative flow chart of Th1, Th2 and Th17 cells in the lung tissues. (**C**) Tregs cells within CD25^+^ cells were identified based on their expression of CD4^+^. (**D**) Representative flow chart of Treg cells in the lung tissues. (**E**–**H**) Percentages of Th1 (**E**), Th2 (**F**), Th17 (**G**) and Treg (**H**) cells in lung tissues of control, RSV and C5aRA-RSV mice. ^*^P < 0.05, ^**^P < 0.01, ^***^P < 0.001 vs. control; ^#^P < 0.05, ^##^P < 0.01, ^###^P < 0.001 vs. RSV 7d. (**I**–**L**) Percentages of Th1 (**I**), Th2 (**J**), Th17 (**K**) and Treg (**L**) cells in lung tissues of control, asthma, RSV-asthma, C5aRA-asthma and C5aRA-RSV-asthma mice. ^*^P < 0.05, ^**^P < 0.01, ^***^P < 0.001 vs. control; ^#^P < 0.05, ^##^P < 0.01, ^###^P < 0.001 vs. asthma; ^&^P < 0.05, ^&&^P < 0.01, ^&&&^P < 0.001 vs. RSV-asthma.

To illuminate C5a-C5aR participates the pathgenic processes of RSV-infected asthma mice whether via regulating CD4^+^T cells immune responses or not, the percentages of Th1, Th2, Th17 and Treg cells in lung tissues were detected. As Fig. 5 shown, the percentages of Th1, Th2, and Th17 cells were significantly increased while the percentages of Treg cells was significantly decreased in RSV-infected mice and asthma mice compared with control mice. Besides, RSV infection could further increase the percentages of Th1, Th2, and Th17 cells but reduce the percentage of Treg cells in RSV-infected asthma mice. Interestingly, C5aRA could overcome adverse changes of the percentages of Th1, Th2, Th17 and Treg cells in RSV-infected, asthma and RSV-infected asthma mice (Fig. 5).

**Figure 6:**
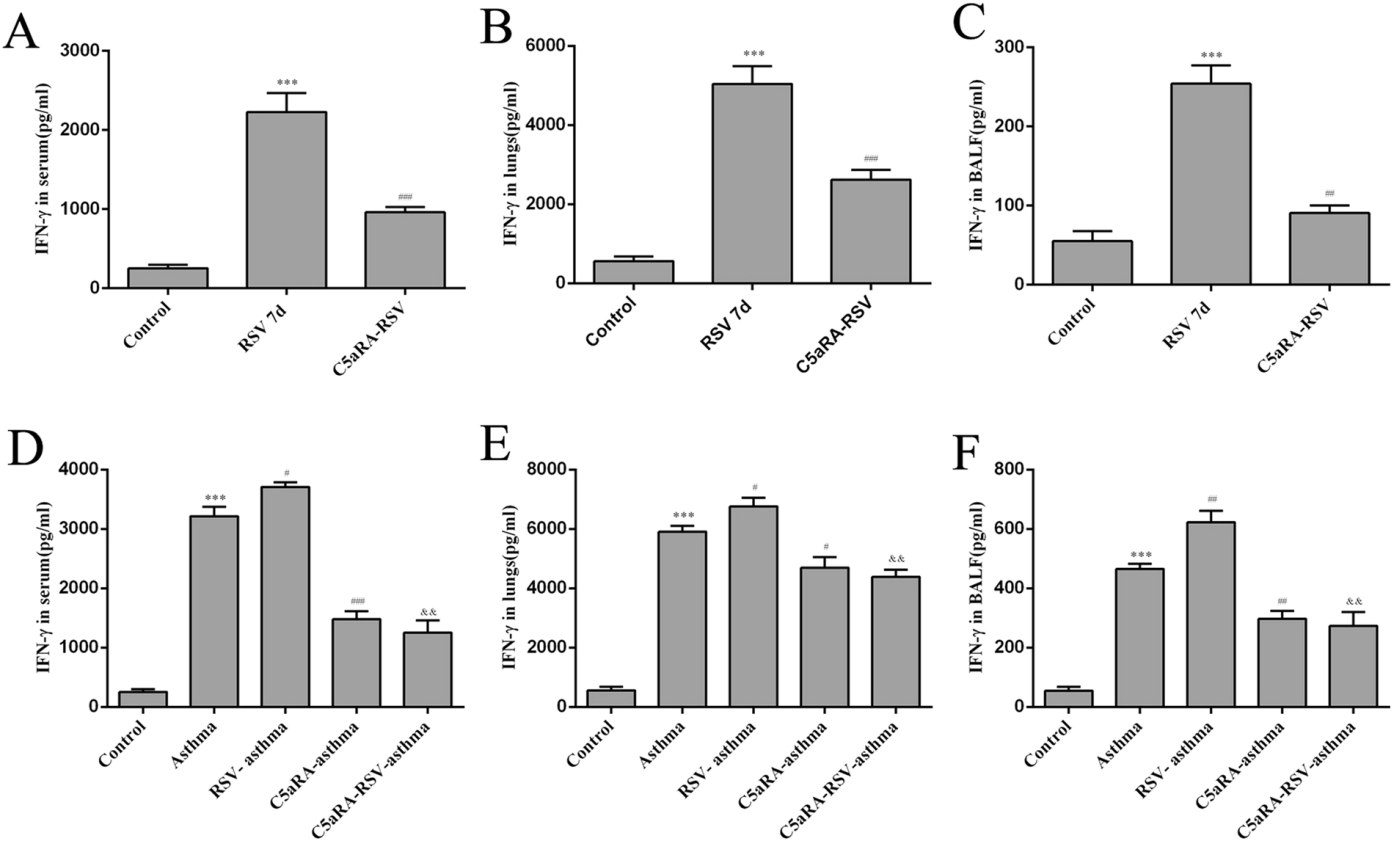
The levels of IFN-γ. (**A**–**C**) Serum IFN-γ levels (**A**), Lung tissue IFN-γ levels (**B**) and BALF IFN-γ levels (**C**) in control, RSV and C5aRA-RSV mice. ^*^P < 0.05, ^**^P < 0.01, ^***^P < 0.001 vs. control; ^#^P < 0.05, ^##^P < 0.01, ^###^P < 0.001 vs. RSV 7d. (**D**–**F**) Serum IFN-γ levels (**D**), Lung tissue IFN-γ levels (**E**) BALF IFN-γ levels (**F**) in control, asthma, RSV-asthma, C5aRA-asthma and C5aRA-RSV-asthma mice. ^*^P < 0.05, ^**^P < 0.01, ^***^P < 0.001 vs. control; ^#^P < 0.05, ^##^P < 0.01, ^###^P < 0.001 vs. asthma; ^&^P < 0.05, ^&&^P < 0.01, ^&&&^P < 0.001 vs. RSV-asthma.

**Figure 7:**
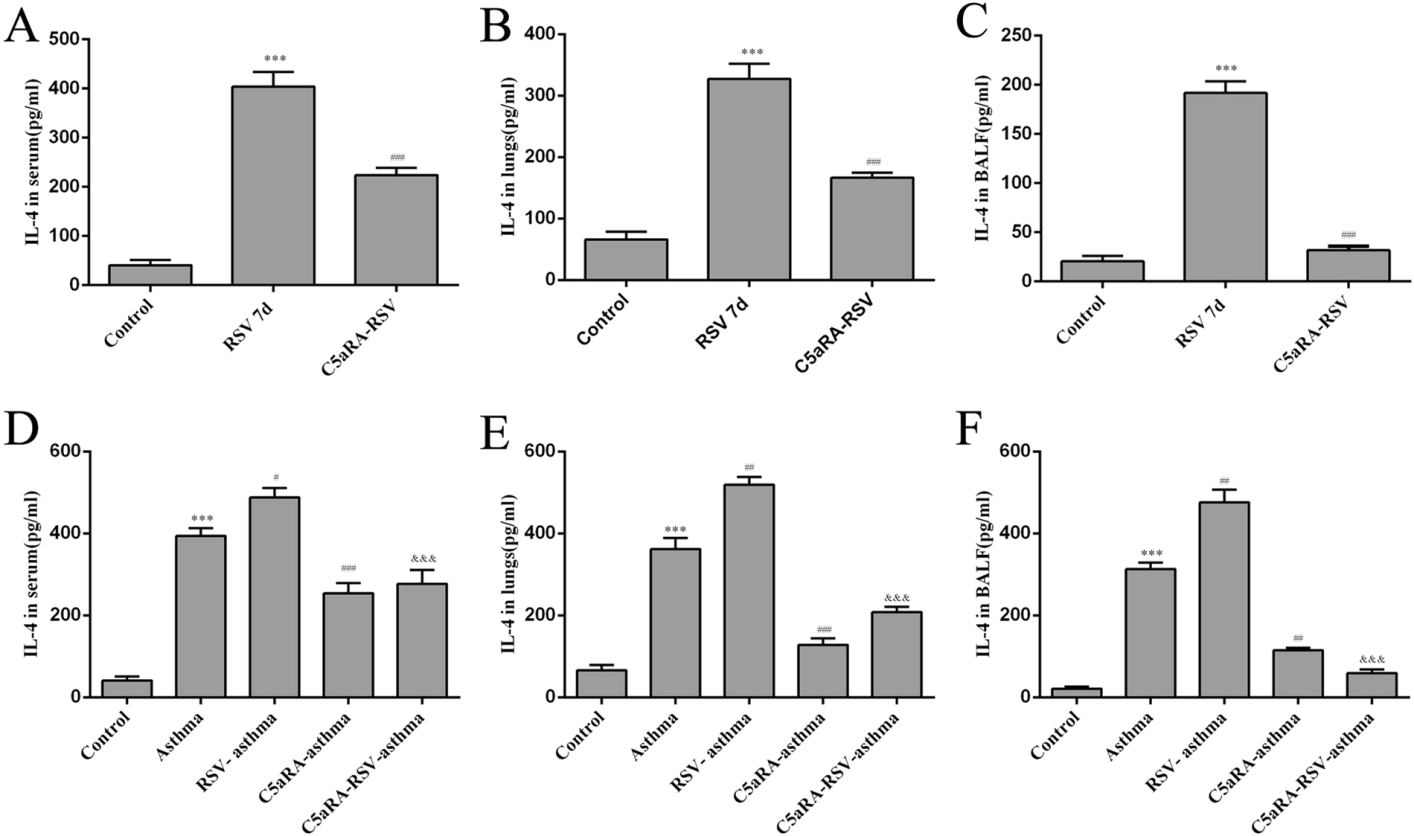
The levels of IL-4. (**A**–**C**) Serum IL-4 levels (**A**), Lung tissue IL-4 levels (**B**) and BALF IL-4 levels (**C**) in control, RSV and C5aRA-RSV mice. ^*^P < 0.05, ^**^P < 0.01, ^***^P < 0.001 vs. control; ^#^P < 0.05, ^##^P < 0.01, ^###^P < 0.001 vs. RSV 7d. (**D**–**F**) Serum IL-4 levels (**D**), Lung tissue IL-4 levels (**E**) and BALF IL-4 levels (**F**) in control, asthma, RSV-asthma, C5aRA-asthma and C5aRA-RSV-asthma mice. ^*^P < 0.05, ^**^P < 0.01, ^***^P < 0.001 vs. control; ^#^P < 0.05, ^##^P < 0.01, ^###^P < 0.001 vs. asthma; ^&^P < 0.05, ^&&^P < 0.01, ^&&&^P < 0.001 vs. RSV-asthma.

**Figure 8:**
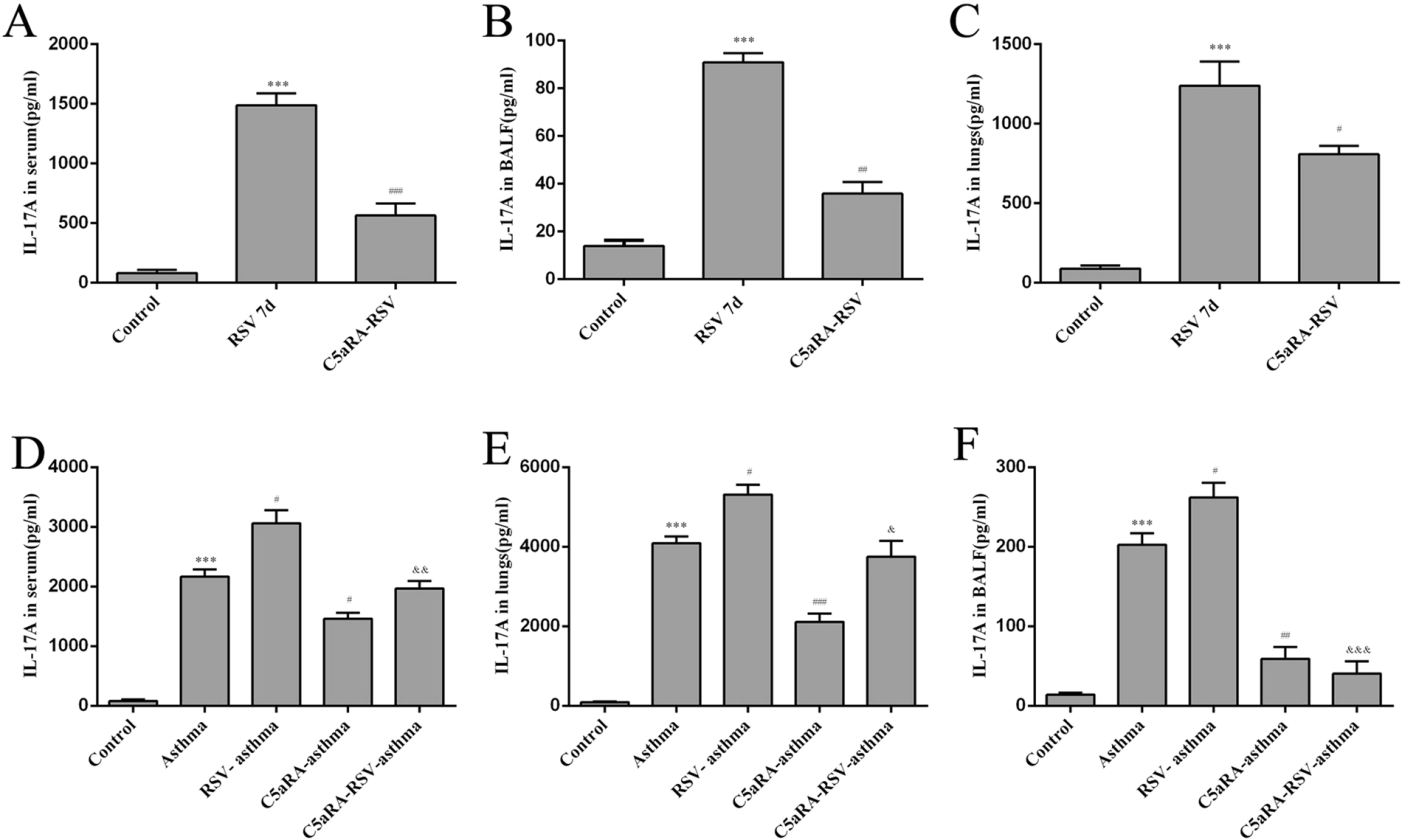
The levels of IL-17A. (**A**–**C**) Serum IL-17A levels (**A**), Lung tissue IL-17A levels (**B**) and BALF IL-17A levels (**C**) in control, RSV and C5aRA-RSV mice. ^*^P < 0.05, ^**^P < 0.01, ^***^P < 0.001 vs. control; ^#^P < 0.05, ^##^P < 0.01, ^###^P < 0.001 vs. RSV 7d. (**D**–**F**) Serum IL-17A levels (**D**), Lung tissue IL-17A levels (**E**) and BALF IL-17A levels (**F**) in control, asthma, RSV-asthma, C5aRA-asthma and C5aRA-RSV-asthma mice. ^*^P < 0.05, ^**^P < 0.01, ^***^P < 0.001 vs. control; ^#^P < 0.05, ^##^P < 0.01, ^###^P < 0.001 vs. asthma; ^&^P < 0.05, ^&&^P < 0.01, ^&&&^P < 0.001 vs. RSV-asthma.

**Figure 9:**
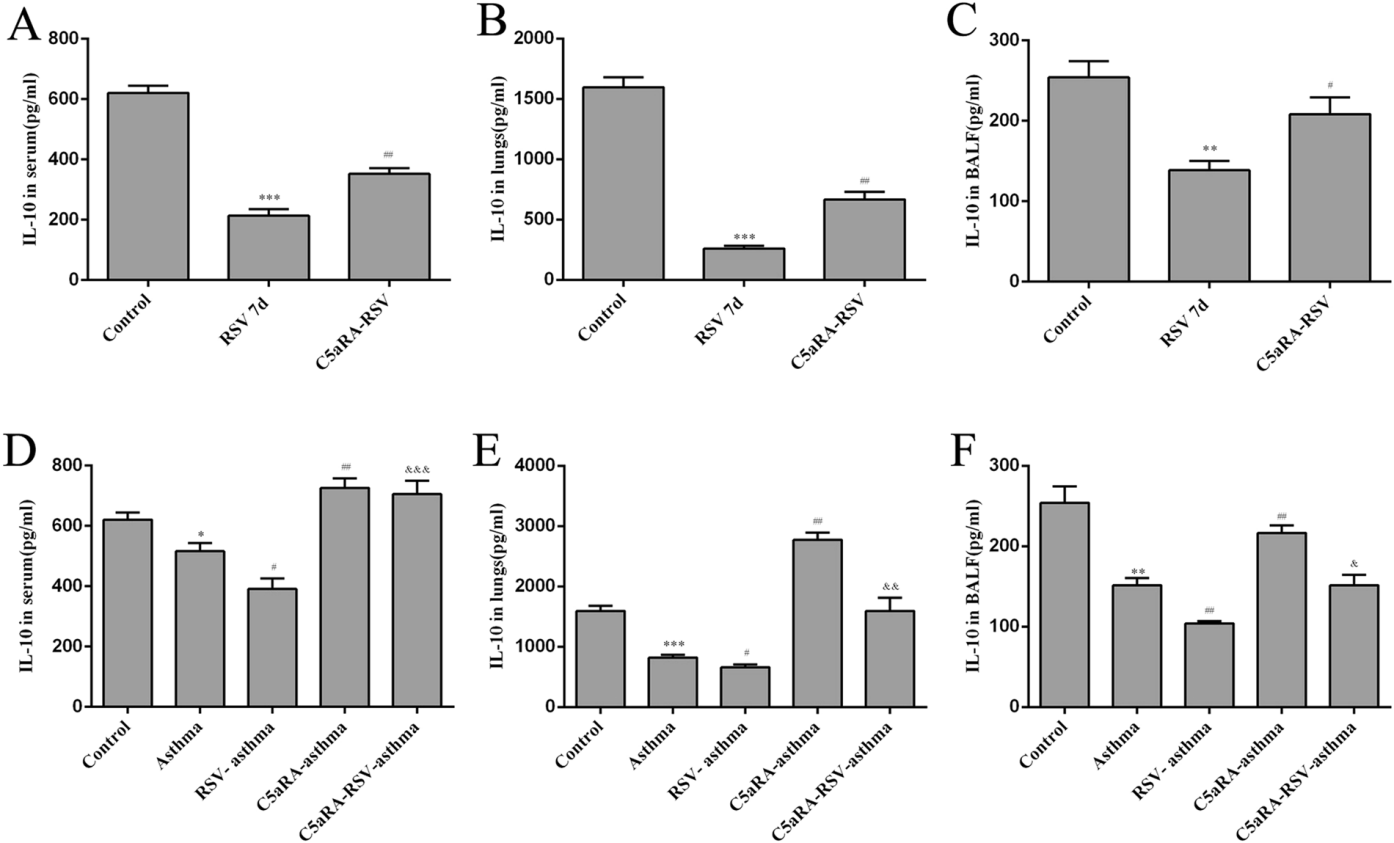
The levels of IL-10. (**A**–**C**) Serum IL-10 levels (**A**), Lung tissue IL-10 levels (**B**) and BALF IL-10 levels (**C**) in control, RSV and C5aRA-RSV mice. ^*^P < 0.05, ^**^P < 0.01, ^***^P < 0.001 vs. control; ^#^P < 0.05, ^##^P < 0.01, ^###^P < 0.001 vs. RSV 7d. (**D**–**F**) Serum IL-10 levels (**D**), Lung tissue IL-10 levels (**E**) and BALF IL-10 levels (**F**) in control, asthma, RSV-asthma, C5aRA-asthma and C5aRA-RSV-asthma mice. ^*^P < 0.05, ^**^P < 0.01, ^***^P < 0.001 vs. control; ^#^P < 0.05, ^##^P < 0.01, ^###^P < 0.001 vs. asthma; ^&^P < 0.05, ^&&^P < 0.01, ^&&&^P < 0.001 vs. RSV-asthma.

Relevant cytokines were tested in serum, lung tissues and BALF by ELISA. Serum IFN-γ, IL-4, and IL-17A levels were increased in RSV and asthma mice compared with control mice while IL-10 levels were decreased. In accord with the trend of Th cells described above, serum IFN-γ, IL-4, and IL-17A levels were augmented while IL-10 levels were declined in RSV-infected asthma mice compared with asthma mice. However, the levels of serum IFN-γ, IL-4, and IL-17A levels were increased while the levels of IL-10 were increased by C5aRA treatment in RSV-infected, asthma and RSV-infected asthma mice. Similar changes were found in lung tissue and BALF IFN-γ, IL-4, IL-10, and IL-17A levels (Figs 6–9).

## Discussion

In the present study, we investigated the role of C5a-C5aR in RSV-infected, asthma and RSV-infected asthma mice. Our data demonstrated that C5aR expressions were higher in airway epithelium, submucosa, smooth muscle, parenchyma and inflammatory cells in RSV-infected mice compared with control mice, while C5aRA could alleviate RSV fusion protein deposition and lung tissue inflammation in RSV-infected mice. These data imply a pathogenic impact for C5a-C5aR in acute RSV infection. Furthermore, C5aR expressions were elevated in RSV-infected asthma mice compared with asthma mice, and C5aRA relieved lung damages, goblet cell metaplasia and RSV fusion protein deposition in RSV-infected asthma mice. C5a levels in serum, lung tissue and BALF were consistent with the expressions of C5aR protein in lung tissues. Taken together, these findings suggest that C5a-C5aR activated by RSV is involved in the pathology of asthma exacerbation.

Immune responses to RSV infection have been extensively researched during the last decades. Evidence suggests that T cell responses are crucial determinants of the outcome of RSV infection as they establish a balance between immunopathology and viral clearance^26^. Following RSV infection, Th1 and Th2 cells activate a cellular immune response, and these Th cells and the cytokines they produce antagonize each other^27,28^. Furthermore, Th17 cells induce mucus production, increase neutrophilic infiltration in lungs, enhance Th2 cytokine generation and attenuate CD8^+^T cell responses^29,30^. In contrast, Treg cells have an anti-inflammatory function, as they suppress pathogenic T cell responses, inhibit lung eosinophilia and protect the immune privilege of self-antigens^31,32^. Consistent with these findings, the present study also showed that the percentages of Th1, Th2, and Th17 cells increased, while the percentage of Treg cells decreased in RSV-infected mice. Concurrent with these changes, levels of IFN-γ, IL-4, and IL-17A in serum, lung tissue and BALF increased in RSV-infected mice but levels of IL-10 declined.

As severe RSV infection has been associated with an increased risk for developing asthma, it is likely that CD4^+^T cells have a role in the immune mechanisms involved in asthma. In fact, evidence suggests that Th1, Th2 and Th17 cells have a proinflammatory impact while Treg cells have a protective effect^1,33^. Previous studies have established that an imbalance in the ratio of FOXP3^+^Treg/Th17 cells results in immune system dysfunction that contributes to the pathogenesis of asthma^34,35^. Assuming the diverse functions of Th1, Th2, Th17 and Treg cells in RSV infection and asthma, we speculated that these CD4^+^T cells also contribute to RSV-induced asthma exacerbation. To confirm this supposition, we established RSV-infected asthma mice. Our results showed that the percentages of Th1, Th2 and Th17 cells increased while the percentage of Treg cells decreased in RSV-infected asthma mice compared with asthma mice. Concurrently, levels of IFN-γ, IL-4, and IL-17A in serum, lung tissues, and BALF increased in RSV-infected asthma mice while levels of IL-10 declined. These findings suggest that RSV-induced asthma exacerbation is associated with an imbalance of Th1, Th2, Th17 and Treg cells and their related cytokines.

Lajoie *et al*. reported that IL-17A production by the IL-23-Th17 axis was mediated by C3a in severe asthma^36^. Schmudde *et al*. indicated that differentiation of Th17 cells, development of maladaptive Th2 and Th17 immunity and T-cell survival in allergic asthma is controlled by C5aR^37^. Above researches pointed out complement activation participated asthma pathogenic processes via regulating CD4^+^T cells immune responses. Besides, Bera *et al*. presented that C3aR deficiency could reduce lung inflammation and accelerate viral clearance through lessening IL-17A levels and mucus production^14^. Up to now, there is no research focusing on the mechanism of C5a-C5aR mediated by RSV infection, meanwhile this project was the first time to report RSV infection exacerbates asthma pathogenesis via activating C5a-C5aR and then regulating CD4^+^T cells immune response.

In the current study, our data indicated RSV infection could induce C5a levels and C5aR expressions in RSV-infected mice, and the degree of C5a levels and C5aR expressions was even more enhancive in RSV-infected asthma mice compared with asthma mice. Additionally, lung damages and RSV fusion protein deposition could be prevented by C5aRA in RSV-infected, asthma and RSV-infected asthma mice. Notably, the number of Th1, Th2, and Th17 cells was decreased while the number of Treg cells was increased in C5aRA-treated RSV-infected asthma mice compared with RSV-infected asthma mice. Moreover, the levels of IFN-γ, IL-4, and IL-17A in serum, lung tissue, and BALF of C5aRA-treated RSV-infected asthma mice were also decreased, while the levels of IL-10 were increased.

In summary, these data indicates that RSV infection could apparently stimulate C5a levels and C5aR expressions in the pathogenesis of RSV-infected asthma mice, meanwhile C5a-C5aR regulates the proportions of Th1, Th2, Th17 and Treg cells, and the immune responses they modulate. Of note, as C5aRA could reverse some of pathology and CD4^+^T cells immune changes in RSV-infected asthma mice, C5a-C5aR may represent a potential therapeutic target in the treatment of this condition, and the further underlying mechnisms demand more exploration.

## Electronic supplementary material

Supplementary Information
