# Supplementary material for: Respiratory Syncytial Virus Exacerbates OVA-mediated asthma in mice through C5a-C5aR regulating CD4+T cells Immune Responses
Source: Sci Rep. 2017 Nov 9;7:15207. doi: 10.1038/s41598-017-15471-w (PMC5680322; doi:10.1038/s41598-017-15471-w)
Supplement: Supplementary file 1 — Supplementary Information [file 41598_2017_15471_MOESM1_ESM.pdf]

Respiratory Syncytial Virus Exacerbates OVA-mediated asthma in mice through C5a-C5aR regulating CD4^{+}T cells Immune Responses

Xinyue Hu[1*], Xiaozhao Li[2*], Chengping Hu[1], Ling Qin[1], Ruoxi He[1], Lisha Luo[1], Wei Tang[1], Juntao Feng[1▲]

Institutional affiliation:

[1]Department of Respiratory Medicine (Department of Respiratory and Critical Care Medicine), Key Cite of National Clincial Research Center for Respiratory Disease, Xiangya Hospital, Central South University, Changsha, Hunan, 410008, China; [2]Department of Nephrology, Xiangya Hospital, Central South University, Changsha, Hunan, 410008, China.

* Co-first author.

Corresponding author information:

Juntao Feng. Department of Respiratory Medicine (Department of Respiratory and Critical Care Medicine), Key Cite of National Clincial Research Center for Respiratory Disease, Xiangya Hospital, Central South University, Changsha, Hunan, 410008, China. E-mail: 23709007@qq.com. Cellphone: +8613786145799.

Supplementary Figure 1

A

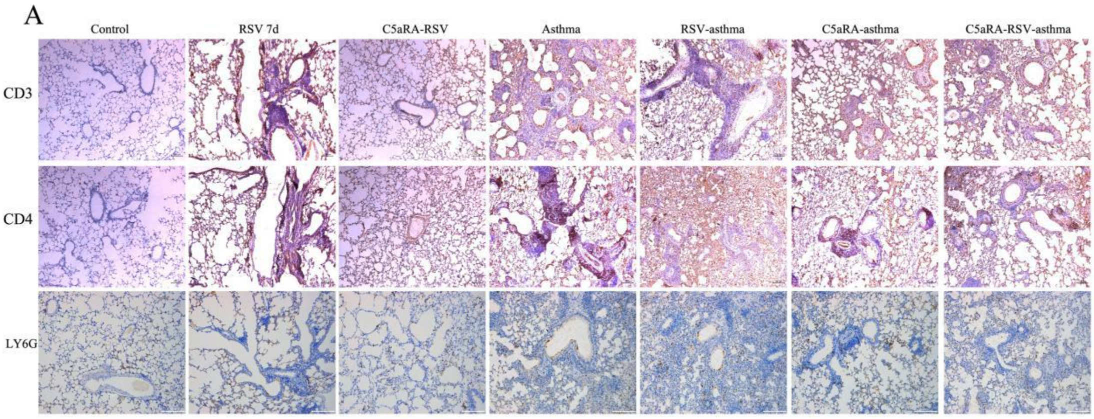

B

Groups	n	Mean IOD
Control	5	0.0081 ± 0.0030
RSV 7d	5	0.0969 ± 0.0247***
C5aRA-RSV	5	0.0430 ± 0.0143*
Asthma	5	0.1642 ± 0.0123***
RSV-asthma	5	0.2047 ± 0.0274
C5aRA-asthma	5	0.0894 ± 0.0083&&
C5aRA-RSV-asthma	5	0.1126 ± 0.00336^^^

C

Groups	n	Mean IOD
Control	5	0.0059 ± 0.0011
RSV 7d	5	0.0908 ± 0.0012***
C5aRA-RSV	5	0.0429 ± 0.0028*
Asthma	5	0.1426 ± 0.0024***
RSV-asthma	5	0.1873 ± 0.0012
C5aRA-asthma	5	0.0967 ± 0.0427&
C5aRA-RSV-asthma	5	0.1029 ± 0.0012^^

D

Groups	n	Mean IOD
Control	5	0.0070 ± 0.0013
RSV 7d	5	0.0892 ± 0.0038***
C5aRA-RSV	5	0.0536 ± 0.0023
Asthma	5	0.1563 ± 0.0236***
RSV-asthma	5	0.1982 ± 0.0035
C5aRA-asthma	5	0.0792 ± 0.0023
C5aRA-RSV-asthma	5	0.1093 ± 0.0164

Supplementary Figure 1 The expressions of CD3, CD4 and LY6G in different mice. (A) Representative images of immunohistochemistry staining for CD3, CD4 and LY6G protein in lung tissues of different mice (100×). (B-D) The semiquantitative of CD3 (B), CD4 (C) and LY6G (D)protein expressions in lung tissues of different mice. $^{*}P<0.05$, $^{**}P<0.01$, $^{***}P<0.001$ vs. control; $^{\#}P<0.05$, $^{\#\#}P<0.01$, $^{\#\#\#}P<0.001$ vs. RSV; $^{\&}P<0.05$, $^{\&\&}P<0.01$, $^{\&\&\&}P<0.001$ vs. asthma; $^{\wedge}P<0.05$, $^{\wedge\wedge}P<0.01$, $^{\wedge\wedge\wedge}P<0.001$ vs. RSV-asthma.

Supplementary Figure 2

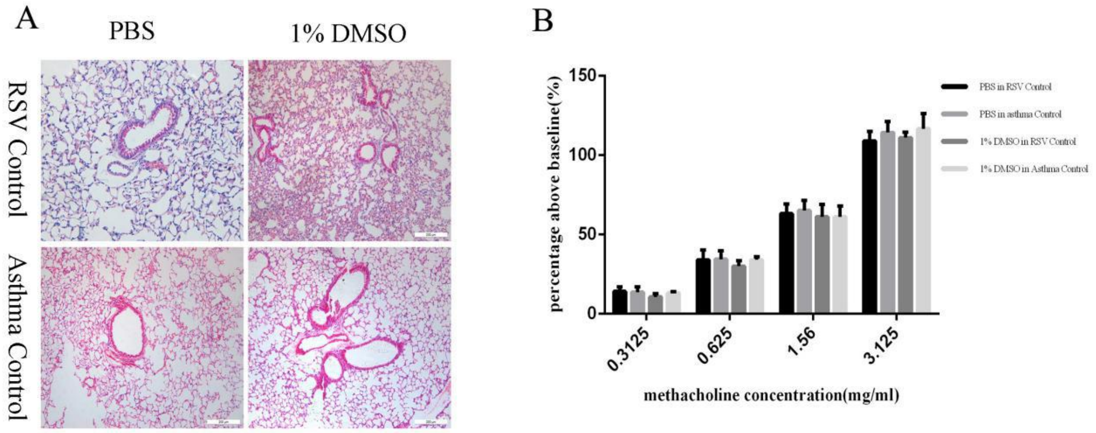

Supplementary Figure 2 The comparison between the impact of 1% DMSO and PBS in normal mice used as control group for RSV and asthma mice. (A) Representative images of H&E staining (100x). (B) AHR. The impact of PBS and 1% DMSO in AHR of normal mice was no significant differences.
